# In Vitro Regeneration of Southern Italian Grapevine Cultivars from Embryogenic Calluses and Protoplasts

**DOI:** 10.3390/plants14213262

**Published:** 2025-10-25

**Authors:** Valeria Ereddia, Chiara Catalano, Fabrizio Salonia, Lara Poles, Edoardo Bertini, Sara Zenoni, Antonio Sparacio, Daniele Oliva, Elisabetta Nicolosi, Alessandra Gentile

**Affiliations:** 1 Department of Agriculture, Food and Environment, University of Catania, Via Santa Sofia 100, 95123 Catania, Italy; valeria.ereddia@phd.unict.it (V.E.); fabrizio.salonia@unict.it (F.S.); elisabetta.nicolosi@unict.it (E.N.); alessandra.gentile@unict.it (A.G.); 2 Agriunitech s.r.l., Via Valdisavoia n. 5, 95123 Catania, Italy; lara.poles@gmail.com; 3 Edivite s.r.l., San Pietro Viminario, Quartiere San Mauro 30, 35020 Padova, Italy; edoardo.bertini@univr.it (E.B.); sara.zenoni@univr.it (S.Z.); 4 Department of Biotechnology, University of Verona, Strada Le Grazie 15, 37134 Verona, Italy; 5 Istituto Regionale del Vino e dell’Olio (I.R.V.O.), Via Libertà 66, 90143 Palermo, Italy; antonio.sparacio@regione.sicilia.it (A.S.); daniele.oliva@regione.sicilia.it (D.O.)

**Keywords:** local varieties, in vitro culture, floral tissue, pollen stage, induction media

## Abstract

Efficiency in vitro regeneration is a crucial prerequisite for the application of New Nenomics Techniques (NGTs) in grapevine (*Vitis vinifera* L.) for improving resistance to biotic and abiotic stresses. This is especially true given that their management must be addressed sustainably, considering the impact of climate change. Unfortunately, in vitro plant regeneration and the establishment of embryogenic calluses are two genotype-dependent processes. Up to now, extensive research has been conducted on major international cultivars, whereas studies on the application of in vitro protocols for autochthonous cultivars remain limited. In this study, protocols for the acquisition of embryogenic calluses were applied on the most relevant Sicilian grapevine cultivars: the red-skinned ‘Frappato’, ‘Nerello mascalese’, and ‘Nero d’Avola’, and the white-skinned ‘Grillo’, ‘Carricante’, and ‘Catarratto’. Stamens and pistils were cultured in two different induction media (PIV and MSII) and at three stages (mother cells in the late premeiotic phase, tetrads, and mature pollen) to induce embryogenic calluses. Five thousand explants per cultivar were cultured, forming calluses in four selected cultivars. Plantlets were successfully generated from calluses of ‘Carricante’, ‘Frappato’, and ‘Nero d’Avola’. Moreover, protoplasts were isolated from ‘Frappato’ and ‘Nero d’Avola’. Our results establish a critical foundation for developing successful regeneration protocols for the future application of NGTs in Sicilian grapevine cultivars.

## 1. Introduction

Grapevine (*Vitis vinifera* L.) is one of the most economically important temperate fruit crops with a global production of 80.1 million tons in 2022 and a harvested area of 7.3 million hectares [1]. Around 50% of the total volume produced is destined for wine production, musts, and juices, while 42% is used to produce table grape [2]. Moreover, grape berries are used in the pharmaceutical industry for the extraction of antioxidants and other beneficial compounds [3]. According to International Organization of Vine and Wine (OIV) statistics, the top three grape-producing countries are China (15.6 million tons), Italy (8.1 million tons), and France (6.2 million tons), followed by the USA, Spain, and Turkey [2]. Grapevine diversity is remarkable, with *V. vinifera* being the major cultivated species [4]; the number of varieties cultivated globally for both wine production and fresh consumption is substantial. For example, Italy cultivates 2441 varieties [5], while France grows 379 varieties [2].

Despite its economic importance, grape cultivation is severely threatened by pathogens and pests, directly impacting yield and fruit quality, while heavy agrochemical use indirectly affects the economic and environmental sustainability of crop management. Moreover, global warming further worsens this situation. Throughout the crop cycle, under mild temperatures and high humidity, almost every organ of the plant is susceptible to the attack of two main pathogens: the oomycete *Plasmopara viticola*, causing downy mildew, and the ascomycete *Erysiphe necator*, causing powdery mildew [6].

In the framework of the Integrated Pest Management approach, crop resistance plays a crucial role by limiting the use of pesticides to a level considered acceptable for human health, non-target organisms, and the environment [7,8]. For this purpose, traditional breeding approaches and genetic transformation have been exploited for the insertion of resistant genes in grape varieties of high commercial value, especially for wine production. Since the success of wine production derives from a complex combination of genotype, environment, and cultural practice, any breeding approach aimed at improving crop resistance towards biotic stress while avoiding the recombination of other traits of interest, thus guaranteeing the cultivar’s identity, is preferred [9]. Moreover, breeding activity could greatly benefit from the use of high-quality germplasm preserved in genebanks, which is maintained in a healthy, virus-free state [10].

New Genomic Techniques (NGTs) include the insertion of a single gene among sexually compatible organisms (cisgenesis) and the accurate modification of specific DNA sequences (genome editing) [11]. These techniques are recognized as useful tools for the improvement of green biotechnology [12], and several experiments utilizing them have been conducted on grape with promising results [13,14,15,16,17,18,19,20,21]. Genome editing techniques allow for fast and accurate genetic modification, including mutation, deletion, and insertion, with the possibility of overcoming the obstacles and the limitations of transgenesis. In knock-out experiments, the application of NGTs for the procurement of plant resistant to major pathogens primarily targets susceptibility genes, rather than those conferring resistance.

Moreover, particularly in Europe, where legislation on Genetically Modified Organisms (GMOs) is still very restrictive, significant efforts have been made to develop techniques that enable genetic modification without introducing exogenous DNA into the organism. A strategy proposed by Olivares et al. consists of the application of geminivirus-derived replicons to mediate CRISPR/Cas9 genome editing on the cultivar ‘Thompson seedless’ to obtain edited plants without the integration of exogenous DNA [13]. More recently, with the same aim, Moffa et al. developed a strategy based on the Cre-lox recombinant technology [22]. However, the approach most commonly used for this purpose is the direct delivery of Cas9 ribonucleoproteins (RNPs) in isolated protoplasts [23]. Protoplasts editing also overcomes the limit of chimerism, because the regeneration of the plants occurs through the further division of a single edited cell [14]. The first attempts of this technique were made by Malnoy et al. for the knock-out of *VvMLO-7*, an S-gene related to the susceptibility to powdery mildew, in cv. ‘Chardonnay’, by the polyethylene glycol (PEG)-mediated transfection of protoplasts [15]. The same technique was then applied by several other researchers in recent years, while some improvements, consisting of the delivery of the Cas9 RNPs by lipofectamine, were performed [20].

In particular, the obtainment of embryogenic calluses and the in vitro regeneration process, while ensuring high efficiency and uniformity in genome editing and minimizing the occurrence of chimeras, also represent significant challenges to the success of these techniques. Also, these challenges are aggravated by the genotype-dependency of the protocols. In fact, the induction of embryogenic calluses and their regeneration into whole plants strongly depend on the specific genotype, stage, and type of the starting plant material, and the hormonal composition of the culture medium [24]. As a result, ad hoc protocols often need to be developed for each species and cultivar [25,26,27].

In several studies on grape, embryogenic callus induction was conducted using floral explants (stamens and pistils) cultured in a solid medium [28,29]. Some exceptions include the use of stamen filaments cultured in a liquid medium [30], and the culture of whole flowers [31]. In Maillot et al., nodal explants were used for the induction of embryogenic calluses [32], while unopened leaves or buds were used by other authors, including Dhekney et al., Najafi et al. [17,33], Li et al., and Olivares et al. [13,34].

In setting up reliable protocols for in vitro regeneration as a preliminary step for the application of NGTs, most research has concentrated on international and widely spread varieties, such as ‘Cabernet Sauvignon’, ‘Chardonnay’, ‘Crimson seedless’, ‘Merlot’, ‘Neo Muscat’, ‘Sangiovese’, ‘Sugraone’, and ‘Thompson seedless’ [13,15,17,18,35,36,37,38,39,40,41]. However, focusing on autochthonous cultivars provides an opportunity to enhance the value of unique local varieties and to preserve regional germplasm. Although not widely recognized on a global scale, grape varieties from Sicily, in the South of Italy, are renowned for producing high-quality wines, many of which carry the European Protected Designation of Origin (PDO) certification. Among these, the DOCG-certified ‘Cerasuolo di Vittoria’, made from the cultivar ‘Frappato’, is a great example of these local cultivars’ potential [42]. Unfortunately, limited research has been conducted on setting up embryogenic callus induction and regeneration protocols for Sicilian grape cultivars. Recently, Catalano et al. reported the efficiency of embryogenic callus induction in three Sicilian grape varieties: ‘Catarratto’, ‘Nero d’Avola’, and ‘Frappato’ [42]. Floral explants were cultured in MS medium with three different combinations of plant growth regulators. The induction efficiency reached in this study was between 2.8% and 6.7%, and the authors focused on the ploidy alteration of the plants regenerated in vitro as a useful resource, in terms of increased variability, for different quality traits.

Given the strong genotype dependency of in vitro regeneration protocols and the critical role of both tissue type and developmental stage of starting material, this study aims to establish efficient protocols for in vitro regeneration and protoplast isolation in six representative Sicilian grapevine cultivars by identifying the most suitable combinations of culture media, pollen developmental stages, and floral tissues. The optimization of in vitro regeneration methodologies would provide a solid basis for the application of NGTs in the genetic improvement of Sicilian autochthonous varieties, ultimately supporting their conservation, enhancement, and valorization within the regional context.

## 2. Results

### 2.1. Induction of Embryogenic Calluses

**Figure 1:**
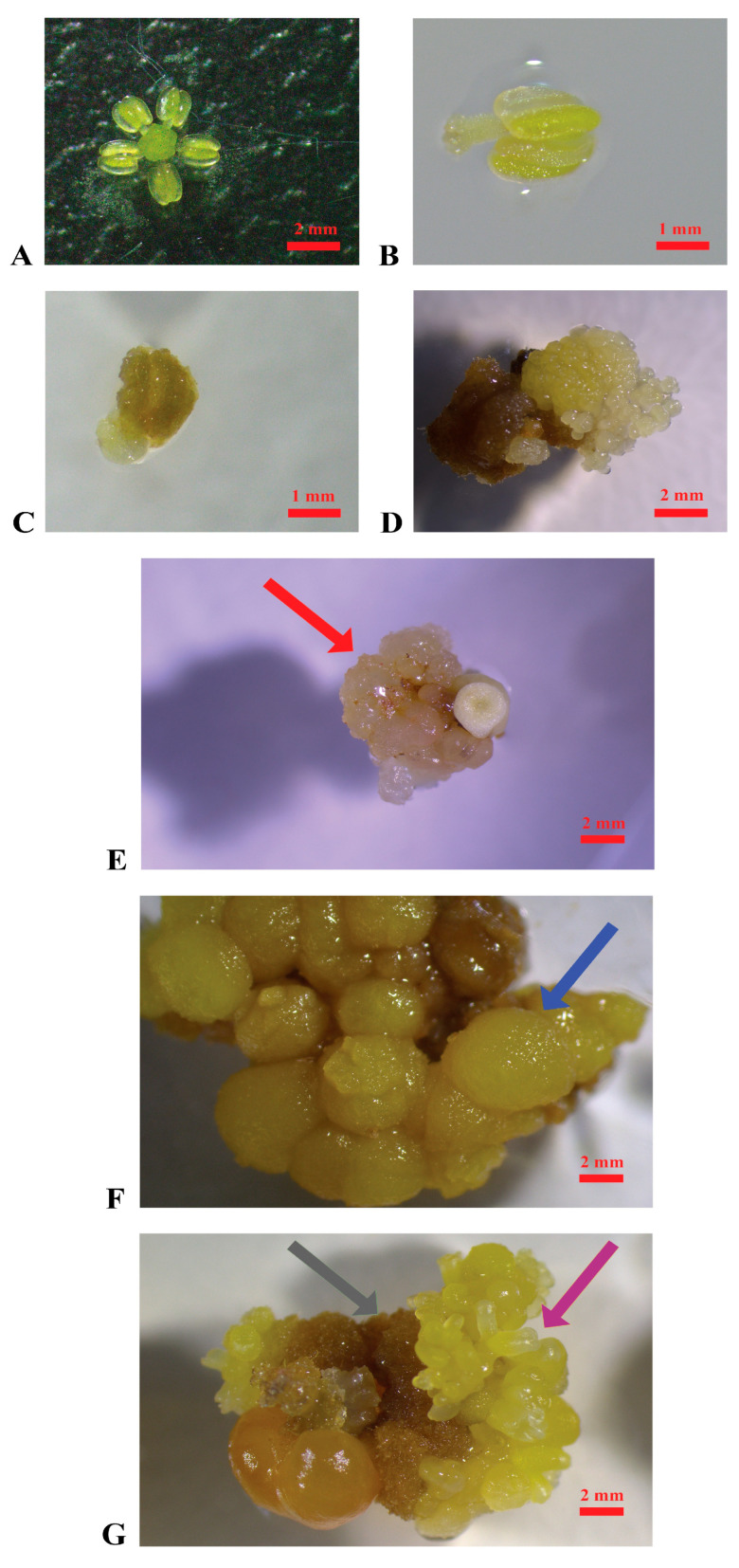
Induction of embryogenic calluses. (**A**): Stamen and pistil of ‘Nero d’Avola’ before cultivation; (**B**): stamen of ‘Catarratto’ immediately after the cultivation in PIV medium; (**C**): stamen of ‘Grillo’ after one month of cultivation in MSII medium; (**D**): stamen of ‘Nero d’Avola’ producing embryogenic calluses (3 months after cultivation) in MSII medium; (**E**): embryogenic calluses of ‘Nerello mascalese’ in the propagation medium C1P indicated by the red arrow; (**F**): non-embryogenic calluses of ‘Carricante’ cultured in C1P medium indicated by the blue arrow; (**G**): brown calluses and embryos of ‘Nero d’Avola’ cultured in MSII medium indicated by the grey and violet arrows, respectively (scale bars = 2 mm).

**Figure 2:**
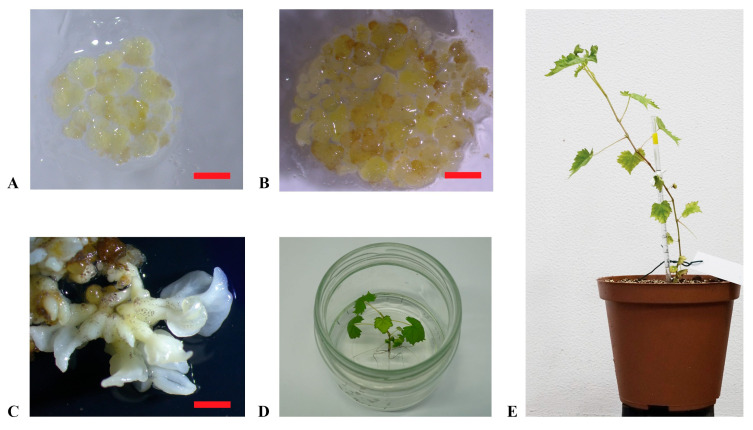
Plant regeneration from embryogenic calluses. (**A**,**B**): embryogenic calluses of ‘Nero d’Avola’ and ‘Frappato’, respectively, cultured in the propagation medium C1P; (**C**): embryos developed from calluses of ‘Carricante’ in X6 medium; (**D**): regenerated plant of ‘Carricante’ cultured in the regeneration medium; (**E**): plant of ‘Nero d’Avola’ regenerated from embryogenic calluses (scale bars = 2 cm).

In Figure 1 and Figure 2, the different stages of the induction of embryogenic calluses from grape flower tissues and plant regeneration are summarized.

A total of 5000 explants were cultured per variety (Figure 1A,B) as specified in the Section 4. The appearance of embryogenic calluses was first observed after three months of cultivation in the induction media MSII and PIV (Figure 1C–E). Overall, several changes occurred in the explants during cultivation: within the first month, most of them turned brown and increased in size; later, several produced calluses with different characteristics (watery, hard, brownish) and even embryos (Figure 1E–G).

In Table 1, the efficiency of callus induction is reported per cultivar, considering as variables the induction medium used (PIV and MSII), the pollen stage (mother cells in the late premeiotic phase, tetrads, mature pollen), and the flower tissue (pistils or stamens). The highest efficiency in embryogenic callus induction was registered in pistils of ‘Nero d’Avola’, followed by ‘Carricante’ and ‘Catarratto’, when cultured at the mother cell stage in the late premeiotic phase in MSII induction medium, with percentages of 8.0%, 7.3%, and 2.9%, respectively. ‘Grillo’ registered the production of a minimum quantity of calluses (0.65%) when stamens were cultured at the mother cell stage in MSII medium. Embryogenic calluses were obtained from ‘Nerello mascalese’ only in the case of stamens cultured at the mother cell stage in the PIV medium (0.32%), while in all the other media–pollen stage–flower tissue combinations, explants appeared necrotic (Supplementary Figure S1). ‘Frappato’ explants demonstrated a callus induction efficiency of 1.5% from stamens at the tetrad stage cultured in MSII. Overall, MSII performed as the best embryogenic callus induction medium in comparison with PIV, reporting an average of 1.98% (±0.45%) and 0.36% (±0.11%), respectively, with a statistically significant difference (Student’s *t*-test *p* value < 0.01). In Supplementary Figure S1, explants from various grapevine cultivars are shown after two months of culture in the two different induction media, illustrating the distinct responses associated with each cultivar–medium interaction.

When subcultured in the propagation medium (C1P), ‘Catarratto’ and ‘Carricante’ calluses degenerated within a few months, with consistent changes in aspect and texture, while ‘Grillo’ and ‘Nerello mascalese’ did not survive, developing necrosis. ‘Nero d’Avola’, ‘Carricante’, and ‘Frappato’ calluses, which were of granular texture and white or yellow, were used in regeneration experiments to confirm they were embryogenic (Figure 1D,E). ‘Nero d’Avola’ and ‘Frappato’ calluses, which performed the best in proliferation, were also used in protoplast isolation experiments.

### 2.2. Plant Regeneration from Embryogenic Calluses

‘Carricante’, ‘Frappato’, and ‘Nero d’Avola’ calluses were used in regeneration experiments through subculture in the X6 medium (Figure 2A,B). Embryos developed in 4–6 weeks from calluses and were transferred into the germination medium to grow into plantlets (Figure 2C,D). This process was entirely successful, resulting in 12, 3, and 4 plantlets obtained from ‘Carricante’, ‘Frappato’, and ‘Nero d’Avola’ calluses, respectively (Figure 2E). In Table 2, regeneration efficiency is reported for the considered accessions in terms of number of embryos or plants obtained per single cultivar per gram of starting embryogenic calluses amount [43]. Despite the high number of embryos obtained, ‘Frappato’ reported a medium–low regeneration of 10 plants per gram of starting embryogenic callus material. A similar result was obtained for ‘Nero d’Avola’, while ‘Carricante’ performed the best (23.3 plants per gram of starting embryogenic masses).

### 2.3. Protoplasts Isolation, Cultivation, and Regeneration

**Figure 3:**
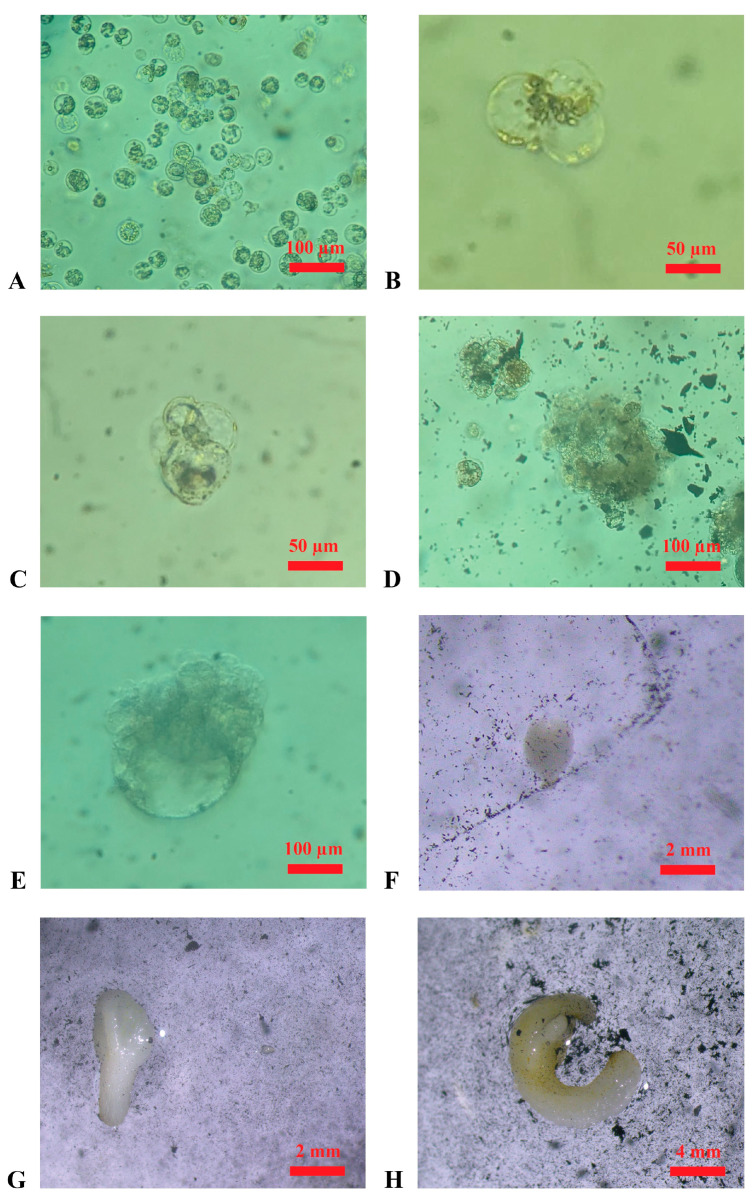
Embryo regeneration from protoplasts in the cultivation medium (in the dark at 25 °C, ±1 °C). (**A**): Protoplasts derived from the enzymatic digestion of embryogenic calluses of ‘Nero d’Avola’ in the wash solution (after filtration); (**B**): first cellular divisions, appearing till ~20 days after protoplast isolation in ‘Nero d’Avola’; (**C**): further cellular divisions appearing ~30 days after protoplast isolation in ‘Frappato’; (**D**): microcolony formation after ~50 days from protoplast isolation in ‘Frappato’; (**E**): globular-stage embryo appearing ~70 days after protoplast isolation in ‘Nero d’Avola’; (**F**,**G**) heart-shape and torpedo-stage embryos after ~80 days of cultivation in ‘Nero d’Avola’; (**H**): embryo obtained after ~90 days of ‘Frappato’ protoplast cultivation.

Protoplast isolation experiments were successful for both ‘Nero d’Avola’ and ‘Frappato’. In two independent experiments, 1.9 × 10^6^ and 2.4 × 10^6^ protoplasts were isolated from calluses of ‘Nero d’Avola’, while 2.6 × 10^6^ and 5.9 × 10^6^ were isolated from ‘Frappato’ calluses. Protoplast isolation using the protocol described in the Materials and Methods yielded positive results; therefore, alternative procedures were not pursued. Cell divisions were first observed 20 days after protoplast isolation (Figure 3B,C), and microcolonies were observed 50 days after isolation (Figure 3D). The regeneration of the first embryo occurred at 3 months after protoplast isolation from both genotypes (Figure 3E,F), and each isolated embryo was then transferred into the germination medium and observed for developmental progress.

## 3. Discussion

In this study, regeneration protocols via calluses and protoplast systems were employed for Sicilian grapevine cultivars with the ultimate aim of enabling the application of New Genomic Techniques (NGTs) for autochthonous germplasm.

The cultivars under study were six of the most used in Sicilian vineyards: ‘Nero d’Avola’, ‘Nerello Mascalese’, and ‘Frappato’, as red-berry varieties and ‘Catarratto’, ‘Carricante’, and ‘Grillo’ as white-berry varieties. Stamens and pistils were used as the starting material (Figure 1A,B), as in most of the research conducted in this field. Floral tissues were cultured at three distinct stages of pollen development (mother cells in the late premeiotic phase, tetrads, mature pollen) to account for any potential difference among varieties and stages of the collected material for callus induction experiments. Taking all these variables into account, only two callus induction media were tested, including one commonly used in the literature (PIV) [29], and another less frequently employed but characterized by a distinct hormonal composition that appears nonetheless promising (MSII) [44]. Embryogenic callus induction had positive results in ‘Nero d’Avola’, ‘Carricante’, ‘Catarratto’, and ‘Frappato’. Specifically, ‘Catarratto’ and ‘Nero d’Avola’ showed callus induction efficiencies of 2.88% and 7.95%, respectively, under the best conditions (Table 1). These results are comparable to, or even higher than, those reported by Catalano et al. [42], who obtained 2.9% and 2.8% for the same cultivars. In contrast, for ‘Frappato’, the callus induction efficiency observed in this study (1.5%) was lower than that reported previously (6.7%). These variations can be attributed to the different hormone combinations used in Catalano et al. [42] (forchlorfenuron—CPPU, 2,4-dichlorophenoxyacetic acid—2,4-D, 2-naphthoxyacetic acid—NOA, thidiazuron—TDZ and 6-benzylaminopurin—BAP), compared to those employed in our experiments (2,4-D, BAP and NOA), highlighting the cultivar-specific responses to hormone type and to their higher concentration. In particular, ‘Carricante’, ‘Frappato’, and ‘Nero d’Avola’ responded very well throughout the whole process, from embryogenic callus formation to plant regeneration, also displaying acceptable percentages of regeneration efficiency (52.2%, 3.5%, and 26.7%, respectively). Conversely, ‘Carricante’, ‘Catarratto’, ‘Grillo’, and ‘Nerello mascalese’ displayed the lowest embryogenic callus induction efficiencies and frequent occurrence of necrosis phenomenon during both induction and propagation in the C1P medium. These varieties failed in our regeneration experiments, probably due to a combination of genetic and physiological factors [45]. These include their intrinsic low regenerative capacity, with no effective response to hormonal induction in vitro, or the fact that they may metabolize auxins and cytokinins differently, preventing the activation of those cellular programmes required for callus induction and embryogenesis. Notably, MSII medium was revealed to be more suitable than PIV for the induction of embryogenic calluses, likely due to its different auxin–cytokinin ratio (1:1), which has been reported to be more effective than that of other proportions for callus formation [46]. As biochemical and hormone profiling analysis were not performed in our experiments, the hypothesis regarding the role of auxin–cytokinin metabolism in callus induction remains speculative at this stage, and hormone profiling needs to be addressed and better elucidated in future studies. Using the available techniques, our study has defined the potential of Sicilian autochthonous grapevine varieties in in vitro regeneration. These findings may serve as a useful reference for future studies involving recalcitrant cultivars that do not easily reach adequate levels of callus induction. Therefore, in the near future, it will be useful to optimize protocols for those varieties that have shown limited regeneration efficiency. The optimization of this protocol should include histological analysis aimed at determining the embryogenic potential of the calluses. Specifically, the proliferation activity, which is higher in embryogenic calluses than in non-embryogenic ones, is related to cytoplasmic density and the number of nuclei [47]. In fact, this approach could provide valuable insights into the nature of the material obtained. To address necrosis issues, different propagation media could be employed in future studies, such as GS1CA [29]. Otherwise, antioxidant molecules could be incorporated, like citric acid, as already used in ‘Chardonnay’ [27]. Permadi et al. recently reviewed strategies for mitigating the issue of browning in in vitro culture, such as submerging explants in an antioxidant solution to prevent browning or incorporating anti-browning agents into the culture media [48]. Another potential improvement would be the application of protocols that induce secondary embryogenesis [49], which can enhance the quantity of embryogenic material. In our study, this was a limiting factor for the subsequent step of protoplast isolation, which was successfully performed only in ‘Nero d’Avola’ and ‘Frappato’.

Although there is significant awareness of the genotype-dependency of these processes, exploring the genetic basis behind the different behaviours of these varieties remains a challenging and interesting research topic, though limited studies have been conducted in this area. Recently, Nuzzo and collaborators reviewed the mechanisms involved in the regeneration process in both somatic embryogenesis and organogenesis, such as the molecular regulation of regeneration and the genetic variability of regenerants (somaclones and chimera), which still represent a bottleneck in grape genetic improvement [50].

Martínez and colleagues, recently demonstrated the overexpression on *BBM* (*BABY BOOM*) and *VvSERK2* (*Somatic Embryogenesis Receptor Kinase*) genes, both related to embryogenic competence, in cotyledonary somatic embryos that were cultured in the presence of 0.5 mM sodium butyrate, a histone deacetylase inhibitor [47]. These results should be considered in future regeneration experiments, both for refining protocols and for further investigating the genetic determinism of embryogenesis in grapevine.

Overall, our study evidenced the dependency of the embryogenic callus induction process according to the cultivar and the induction media, with MSII performing better than PIV, pollen stage, and flower tissue. According to our results, the best media–tissue–stage combination would enable the acquisition of embryogenic calluses from Sicilian grape varieties of interest, enabling the future application of NGTs in these autochthonous varieties for enhancing resistance to biotic stress and their valorization in the global wine-growing context.

## 4. Materials and Methods

### 4.1. Plant Material and Sterilization

**Figure 4:**
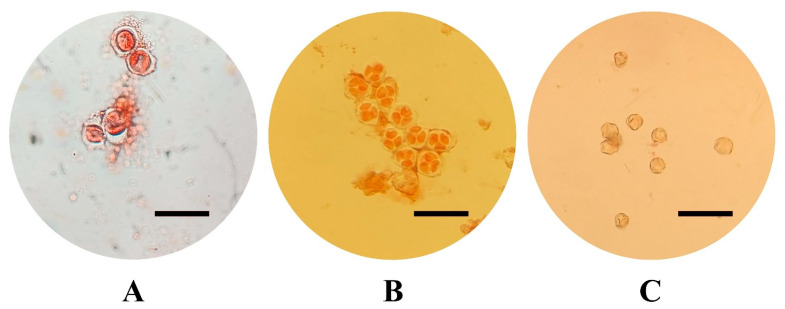
Pollen developmental stage: pollen mother cells with callose wall (**A**), tetrads (**B**), mature pollen (**C**) (acetocarmine staining, scale bars = 50 µm).

The varieties selected for this study, all belonging to *V. vinifera* species, include six of the most economically important in Sicily: three are red-skinned (‘Frappato’, ‘Nerello Mascalese’, and ‘Nero d’Avola’) and three are white-skinned (‘Carricante’, ‘Catarratto’, and ‘Grillo’). For the in vitro cultivation and production of embryogenic calluses, flowers were collected from both fruiting cuttings, cutting collected from adult plant which were forced to flowering in controlled conditions after a vernalization period, [51,52] and from adult plants cultured in vineyards on the north-eastern and eastern slopes of Mount Etna in the province of Catania, in an area particularly suited to viticulture. Five clusters were randomly collected from adult plants in vineyards and from fruiting cuttings in order to (1) isolate flowers at different developmental stages and (2) collect a sufficient number of explants (5000 per cultivar) to be cultured in vitro. At least three distinct clusters were used per cultivar stage of the pollen combination. For each cultivar, 2500 explants were cultured in each induction medium, while around 800 explants were cultured per pollen stage. Floral tissues (stamens and pistils) were cultured at three different developmental pollen stages: mother cells in the late premeiotic phase with callose walls, briefly referred to as mother cells, tetrads, and mature pollen (Figure 4) [18]. Pollen stage was determined by cytological analysis, which involved dissecting the stamens and examining their content under a Leica DM2500 (Leica Microsystems, Wetzlar, Germany) optical microscope under a bright field, after staining with Acetocarmine Solution according to Kultschitzky (TCI Europe N.V., Zwijndrecht, Belgium). In particular, stamens were collected from flowers in different parts of the cluster (basal, middle, and apical) to obtain a reliable estimation of the pollen stage. Once the developmental stage was identified, the sterilization of the collected clusters was performed by immersion for 10 min in a 3% solution of NaClO added with 40 µL of Tween-20 (Sigma-Aldrich, St. Louis, MO, USA), followed by three 5 min washes in sterile distilled water.

### 4.2. Cultivation of Stames and Pistils

Clusters were observed under a Leica EZ4 (Leica Microsystems, Wetzlar, Germany) stereomicroscope and manipulated in sterile conditions to dissect flowers, using two sterile insulin needles. After removing the calyptra, stamens (ensuring that they included the filament and the anther) and pistils were collected and placed into Petri dish plates (Ø 90 mm) following a scheme of 44 stamens and 8 pistils per dish. Stamens and pistils were maintained in the dark at 25 °C (±1 °C) on two different cultivation media to promote embryogenic callus induction: PIV [29] and MSII [44] (Table 3). PIV is one of the most used in regeneration protocols, and its effectiveness is widely recognized [27,31,40,53,54,55]. MSII represents an alternative, characterized by a different ratio of the hormones 6-benzylaminopurin and 2,4-dichlorophenoxyacetic acid (BAP and 2,4-D), considered idoneous for callus proliferation [46], and an additional auxin 2-naphthoxyacetic acid (NOA) (Table 3). Explants were cultured in Petri dish plates (Ø 90 mm) filled with 20 mL media.

### 4.3. Callus Propagation and Regeneration

Explants were monitored monthly under a Leica Ivesta3 (Leica Microsystems, Wetzlar, Germany) stereomicroscope equipped with a camera and propagated in Petri dish plates (Ø 90 mm) filled with 20 mL of C1P medium [58], composed of MS salt and vitamins (Duchefa Farma, Haarlem, The Netherlands), 5 µM 2,4-D (Sigma-Aldrich, St. Louis, MO, USA), 1 µM BAP (Sigma-Aldrich, St. Louis, MO, USA), 3% sucrose (Sigma-Aldrich, St. Louis, MO, USA), and 0.5% phytagel (Sigma-Aldrich, St. Louis, MO, USA) (pH 5.8). A variable number (usually 4 spots consisting of 0.100 g each) of healthy calluses, granular and white or yellow, appearing during proliferation activity, were transferred into fresh C1P medium every four weeks and maintained in the dark at 25 °C (±1 °C).

Callus induction efficiency was calculated considering the ratio between the number of explants showing callus proliferation and the total number of explants cultured per variety, induction media, floral tissue type, and pollen stage. Descriptive statistical analyses (mean, standard deviation, standard error), Student’s *t*-test (*p* value < 0.01), and ANOVA were performed using the ‘stats’ package of R software v. 4.1.0 [59].

We conducted regeneration experiments, cultivating calluses in Petri dish plates (Ø 60 mm) filled with 10 mL of X6 medium [33], comprising MS salt and vitamins (lacking glycine, Duchefa Farma, Haarlem, The Netherlands), 30 mM KNO_3_, 6.81 mM NH_4_Cl, 5.56 mM myo-inositol (Sigma-Aldrich, St. Louis, MO, USA), 6% sucrose (Sigma-Aldrich, St. Louis, MO, USA), 0.05% activated charcoal (Duchefa Farma, Haarlem, The Netherlands), and 0.25% gelrite (pH 5.8, Sigma-Aldrich, St. Louis, MO, USA) in the dark at 25 °C (±1 °C). Regeneration efficiency was estimated considering the initial number of explants producing embryogenic calluses and the number of embryos which developed completely into plantlets. The obtained embryos were transferred into glass boxes (Ø 100 mm, 100 mm H) filled with 50 mL of the germination medium, composed of NN macro, micro, and vitamins (Duchefa Farma, Haarlem, The Netherlands), 3% sucrose (Sigma-Aldrich, St. Louis, MO, USA) and 0.2% gelrite (pH 5.8, Sigma-Aldrich, St. Louis, MO, USA), promoting the development of the embryos into plantlets. The embryos were maintained under light conditions (16 h photoperiod, 65 μmol m^−2^ s^−1^) at 25 °C (±1 °C), until plantlets developed.

The plantlets obtained were transferred to sterilized peat discs in sterile boxes and then progressively acclimatized to in vivo conditions.

### 4.4. Protoplasts Isolation

For protoplast isolation, embryogenic calluses, appearing healthy, granular, and white or yellow, were transferred in fresh C1P medium 7–10 days before the procedure [31]. One gram of callus was digested with 10 mL of enzymatic solution (0.515 M, 535 mOsm/L), comprising 2% *w*/*v* Cellulase Onozuka, 1% *w*/*v* Macerozyme R-10, 0.05% *w*/*v* Pectolyase Y-23, 10 mM CaCl_2_, 5 mM 2-(N-morpholino) methanesulfonic acid (MES), and 0.5 M mannitol (pH 5.7) [60]. All reagents used for preparing the enzymatic solution were obtained from Sigma-Aldrich (St. Louis, MO, USA). The digestion was performed at room temperature in agitation (50 rpm) per 4 h.

The solution was filtered through a nylon filter (60 μm) and centrifuged at 100 rcf for 10 min. The pellet was resuspended in a wash solution composed of 10 mM CaCl_2_ and 0.5 M mannitol (Duchefa Farma, Haarlem, The Netherlands) and centrifuged again, with the same parameter, three times [40].

### 4.5. Protoplasts Cultivation for Somatic Embryogenesis

The isolated protoplasts were cultured at a concentration of 1 × 10^5^ protoplasts/mL using the disc-culture method [60]. Protoplast pellets were resuspended in 1 mL of solid cultivation media and dispensed in the plates. The cultivation media was composed of NN salts and vitamins (Duchefa Farma, Haarlem, The Netherlands), 10µM 1–naphthaleneacetic acid (NAA, Sigma-Aldrich, St. Louis, MO, USA), 2µM 6-benzylaminopurine (BAP, Sigma-Aldrich, St. Louis, MO, USA), 0.3 M glucose (Sigma-Aldrich, St. Louis, MO, USA), 0.09 M sucrose (Sigma-Aldrich, St. Louis, MO, USA), and 0.2% gelrite (Sigma-Aldrich, St. Louis, MO, USA) (pH 5.7). The droplets containing the protoplasts were covered with liquid cultivation media, composed as described above with 0.3% of activated charcoal (Duchefa Farma, Haarlem, The Netherlands) and no gelrite, and maintained in the dark at 25 °C (±1 °C) in Petri dish plates (Ø 60 mm). The liquid media was replaced every two weeks. Embryos developed from protoplasts under regeneration protocols were subcultured as described in the previous paragraphs.

## 5. Conclusions

In this study, we evaluated different combinations of culture media, pollen developmental stages, and floral tissues to optimize embryogenic callus induction and plant regeneration in six valuable Sicilian autochthonous grapevine cultivars. Our results show that MSII medium was the most effective for embryogenic callus induction, while the influence of pollen stage and floral tissue varied depending on the genotype. These findings clearly demonstrate that the protocols can be tailored to each cultivar, thereby achieving the main goal of identifying efficient in vitro regeneration strategies for these grape varieties. Duly, our results can be summarized as follows:

- ‘Nero d’Avola’, ‘Carricante’, ‘Catarratto’, and ‘Frappato’ embryogenic calluses were obtained from pistils and stamens cultured in MSII induction medium, with callus induction efficiency reaching up to 7.95% in ‘Nero d’Avola’;
- ‘Nero d’Avola’, ‘Frappato’, and ‘Carricante’ plants were successfully regenerated from embryogenic calluses, with plant regeneration efficiency reaching up to 52.2% in ‘Carricante’;
- ‘Nero d’Avola’ and ‘Frappato’ protoplasts were successfully isolated with yields reaching up to 5.9 × 10^6^ in ‘Nero d’Avola’.

Further optimization of the protocol is still needed for the callus maintenance and proliferation steps in ‘Carricante’, ‘Catarratto’, ‘Grillo’, and ‘Nerello mascalese’, and regeneration efficiency remains to be investigated from protoplasts. However, our results lay a solid foundation for the in vitro regeneration of autochthonous grapevine varieties, providing the basis for the application of NGTs and enhancing their value as important germplasm for viticulture and enology in Sicily.

## Figures and Tables

**Table 1:** Embryogenic callus induction efficiency in the different Sicilian grape varieties, according to the induction media used, the floral tissue type, and the pollen stage.

Cultivar	Pollen Stage	Percentage of Callus Induction (%)			
		MSII		PIV	
		Stamens	Pistils	Stamens	Pistils
‘Carricante’	mother cells	3.03	7.29	- *	-
	tetrads	0.64	1.00	-	0.96
	pollen	1.92	1.92	0.57	-
‘Catarratto’	mother cells	1.40	2.88	-	-
	tetrads	0.20	-	-	-
	pollen	0.17	0.96	-	-
‘Frappato’	mother cells	0.20	-	-	-
	tetrads	1.50	-	-	-
	pollen	-	-	-	-
‘Grillo’	mother cells	0.65	-	0.16	-
	tetrads	0.60	-	0.12	-
	pollen	-	-	0.17	-
‘Nerello mascalese’	mother cells	-	-	0.32	-
	tetrads	-	-	-	-
	pollen	-	-	-	-
’Nero d’Avola’	mother cells	1.03	7.95	-	-
	tetrads	1.54	2.23	0.17	-
	pollen	1.36	1.25	-	-

*—indicates no calluses obtained, mainly due to necrosis.

**Table 2:** Regeneration efficiency (%) for ‘Carricante’, ‘Frappato’, and ‘Nero d’Avola’.

Cultivar	Total Amount of Starting Embryogenic Calluses (g)	No. Embryos Obtained	No. Embryos per Gram of Starting Embryogenic Calluses	No. Plants Obtained	No. Plants per Gram of Starting Embryogenic Calluses
‘Carricante’	0.516	93	180.2	12	23.3
‘Frappato’	0.301	25	83.1	3	10.0
‘Nero d’Avola’	0.430	5	11.6	4	9.3

**Table 3:** Composition of the induction media used in this study as reported in previous studies [29,44].

	PIV	MSII
MACROELEMENTS	NN [56] *	MS [57] *
MICROELEMENTS	MS *	MS *
VITAMINS	MS *	MS *
SUCROSE **	60 g/L	20 g/L
2,4-DICHLOROPHENOXYACETIC ACID **	4.5 µM	2.5 µM
6-BENZYLAMINOPURINE **	8.9 µM	5 µM
2-NAPHTHOXYACETIC ACID **	none	2.5 µM
pH	5.8	5.8

* Duchefa Farma (Haarlem, The Netherlands). ** Sigma-Aldrich (St. Louis, MO, USA).

## Data Availability

The data are available within the article.

## Supplementary Materials

The following supporting information can be downloaded at https://www.mdpi.com/article/10.3390/plants14213262/s1: Figure S1: Explants (stamens or pistils) from the six Sicilian grapevine cultivars after two months of culture on two different induction media at the tetrads stage. This figure highlights the differential morphological responses and callus formation efficiencies, illustrating the influence of cultivar–medium interactions on in vitro regeneration outcomes.
