# Supplementary material for: In Vitro Regeneration of Southern Italian Grapevine Cultivars from Embryogenic Calluses and Protoplasts
Source: Plants (Basel). 2025 Oct 25;14(21):3262. doi: 10.3390/plants14213262 (PMC12608478; doi:10.3390/plants14213262)
Supplement: Supplementary file 1 [file plants-14-03262-s001.zip › plants-3903995-supplementary.pdf]

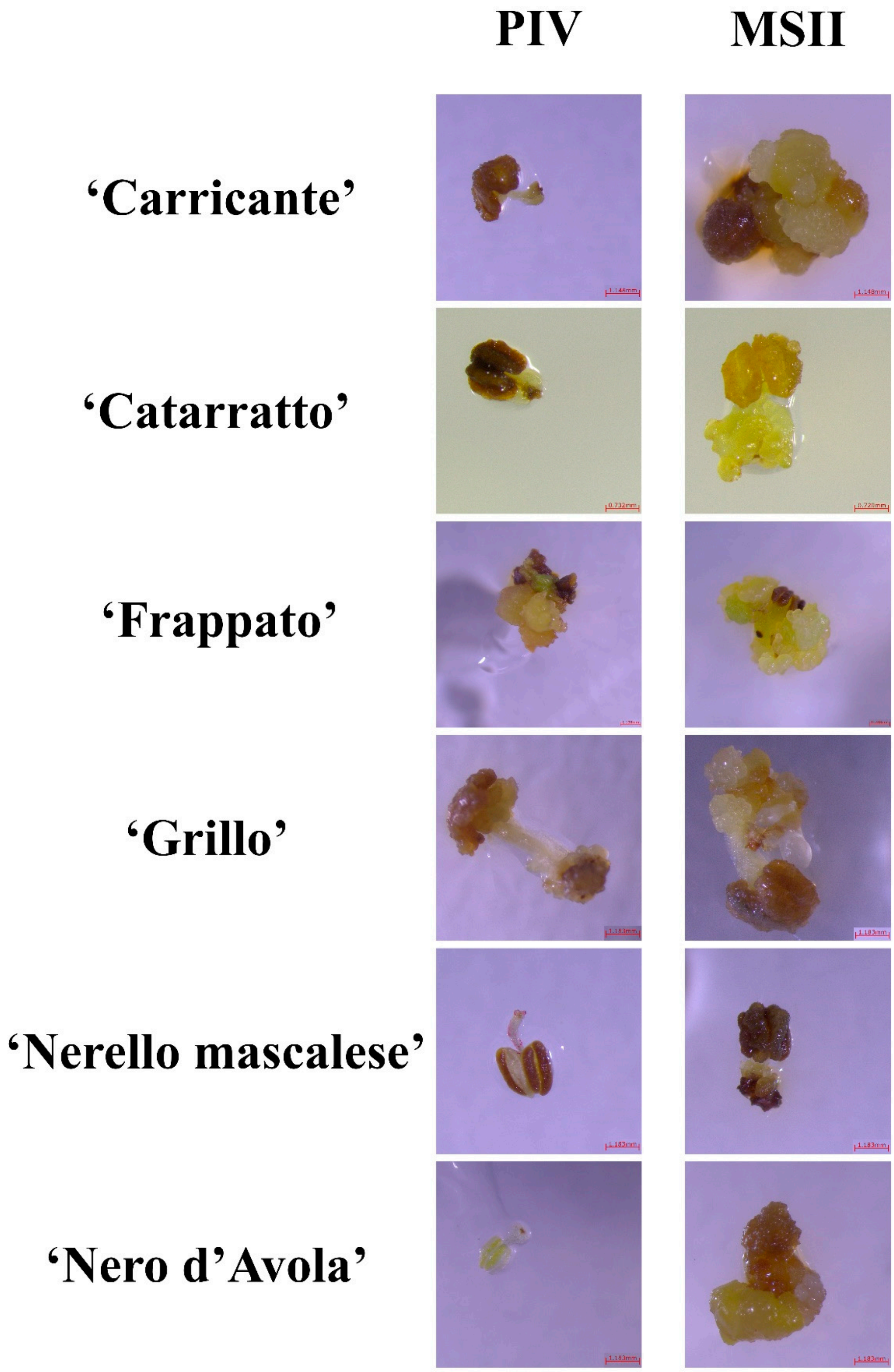

Figure S1. Explants of the six Sicilian grapevine cultivars after two months of culture on the two different induction media at the tetrads stage. Stamens are displayed for ‘Carricante’, ‘Catarratto’, ‘Grillo’, ‘Nerello mascalese’ and ‘Nero d’Avola’, while pistils are displayed for ‘Frappato’. The figure highlights the differential morphological responses and callus formation efficiencies, illustrating the influence of genotype–medium interactions on *in vitro* regeneration outcomes.
